# Mutational landscape of gingivo-buccal oral cancer: new cancer genes and molecular subgroups identified

**DOI:** 10.1186/1755-8166-7-S1-I8

**Published:** 2014-01-21

**Authors:** Partha P Majumder

**Affiliations:** 1 National Institute of Biomedical Genomics, Kalyani, West-Bengal, India

## 

Gingivo-buccal oral cancer (GBOC), an anatomical and clinical sub-type of head and neck squamous cell carcinoma (HNSCC), is prevalent in regions where tobacco-chewing is common. Exome sequencing and other data on 50 GBOC tumor/normal DNA pairs revealed (a) significantly and recurrently mutated genes that are (i) specific (*USP9X*, *MLL4*, *ARID2*, *UNC13C* and *TRPM3*), and (ii) shared with HNSCC (e.g., *TP53*, *CDKN2A*, *PIK3CA*, *HRAS*, *NOTCH1*); (b) new genes with recurrent amplifications (e.g., *DROSHA*, *YAP1*) or homozygous deletions (e.g., *DDX3X*); (c) existence of molecular sub-types, with distinctive mutational profiles; (d) high proportion of C>G transversions, not noted earlier in HNSCC, among tobacco users with high numbers of mutations; and, (e) enrichment of alterations of pathways specific to GBOC, including Neurotrophin signaling, Wnt signaling, dorso-ventral axis formation and axon guidance. Recurrently mutated genes were validated on an independent set of 30 GBOC patients. These findings open new vistas for biological characterization and exploration of therapies.

